# Three-dimensional spatial analysis of temporomandibular joint in adolescent Class II division 1 malocclusion patients: comparison of Twin-Block and clear functional aligner

**DOI:** 10.1186/s13005-023-00404-y

**Published:** 2024-01-06

**Authors:** Yueying Zhang, Jiajing Zheng, Qiuyue Wu, Tianlu Jiang, Hua Xiao, Yusen Du, Yizhe Qi, Zuolin Jin, Feifei Li

**Affiliations:** State Key Laboratory of Oral & Maxillofacial Reconstruction and Regeneration, National Clinical Research Center for Oral Diseases & Shaanxi Clinical Research Center for Oral Diseases, Department of Orthodontics, The Third Affiliated Hospital of Air Force Medical University, Xian, 710032 Shaanxi People’s Republic of China

**Keywords:** Class II malocclusion, Functional orthopedic appliance, Clear aligners, Twin Block, Temporomandibular joint, Cone beam computed tomography

## Abstract

**Background:**

Our study aimed to use three-dimensional (3D) spatial morphological measurement methods to compare the influence of Twin-Block and clear functional aligners on the temporomandibular joint (TMJ) of adolescent Class II division 1 malocclusion mandibular retraction patients. We also aimed to explore the similarities and differences in the effects on the TMJ upon using Twin-Block and clear functional aligner.

**Methods:**

Cone-beam computed tomography (CBCT) data of 49 patients with Class II division 1 malocclusion (Twin-Block group: 24; clear functional aligner group: 25) were collected before and after functional orthodontic treatment, and a 3D model of the TMJ was reconstructed using MIMICS 21.0 software. Eighteen measurement parameters, including the anterior, superior, and posterior joint spaces, were measured and compared using the 3D model.

**Results:**

After the two groups underwent functional appliance treatment, the height, volume, and surface area of the condyle, length of the mandibular ramus and mandibular length increased; The retro-displaced condyle moved to the middle position of the articular fossa, while the rest of the condylar position did not change significantly. Remodeling of the articular fossa after treatment was not evident. The superior joint space of the clear functional aligner group increased, but there was no significant change after Twin-Block appliances treatment.

**Conclusions:**

Both appliances promote condylar growth and sagittal and vertical development of the mandible in adolescent Class II division 1 malocclusion mandibular retraction patients. The length of the mandibular ramus showed a more significant increase following treatment with the Twin-Block appliances than with clear function aligners.

## Background

Class II division 1 malocclusion, which is a common condition encountered in clinical practice, is often characterized by a distal relationship of the molars, labial inclination of the upper anterior teeth, and deep overbite and overjet of the anterior teeth. This condition can seriously affect the facial esthetics, function of the stomatognathic system, and mental health of patients. Class II Division 1 malocclusion has been identified as a possible predisposing factor for traumatic injury involving the maxillary incisors, tooth loss, and frontal facial trauma [1]. Koroluk et al. reported that 29.1% of patients with an overjet ≥ 7 mm already had enamel fractures of the maxillary incisors at the age of 9.8 years [2]. McNamara reported Class II malocclusion to be commonly associated with mandibular retrognathism [3]. Mandibular retrusion and mandibular hypoplasia were reported as the most unacceptable facial features in a previous survey [4].

A two-stage treatment is often used in clinical practice for Class II division 1 malocclusion patients with mandibular retrusion in the growth period. In the first stage, a functional appliance, which may induce mandibular advancement and condylar growth, promote new bone deposition in the temporomandibular fossa, and improve the relationship between the articular disc and fossa [5]. In the second phase, the remaining occlusal problems are corrected.

In cases of orthodontic treatment of patients with mandibular retrusion class II division 1 malocclusion, Twin-Block appliances can decompose the chewing force and transform it into a force conducive to the forward growth of the mandible to promptly correct the sagittal directional disorder of the upper and lower jaws [6]. Although Twin Block has demonstrated significant effects on mandibular advancement, anterior tooth deep overjet reduction, and molar relationship improvement [7], it possesses certain limitations, such as discomfort and unfavorable esthetics and challenges in speech and pronunciation [8]. In recent years, clear functional aligners have gradually gained popularity owing to their superior esthetics and comfort [9]. Previous studies have compared the dental and skeletal effects of using Twin-Block and clear aligners [10, 11]. However, whether clear functional aligners have a significant effect on the growth of the temporomandibular joint (TMJ) remains unclear.

The function orthodontic treatment involves changing the position of the mandible to generate force by the contraction of the relevant muscles; this force is then transmitted to the teeth, skeletal, and TMJ [12]. Change in the mandibular position plays an important role in the treatment with functional appliances and is a key factor in maintaining treatment efficacy. The growth and development of the mandible are influenced by changes in the TMJ [13]. Therefore, the TMJ changes associated with functional orthodontic treatment should be evaluated.

Computed tomography (CT) has transitioned from traditional two-dimensional measurements to three-dimensional spatial analysis [14]. Cone-beam computed tomography (CBCT) has been widely used in clinical practice for nearly 20 years and demonstrates superior three-dimensional (3D) imaging of the teeth and jaws [15]. Studies have reported more accurate evaluation of the structure of the TMJ using 3D spatial measurement [16]. Therefore, this study performed a 3D analysis of the TMJ before and after functional orthodontic treatment and compared the similarities and differences between the effects of Twin-Block and clear functional aligners to provide a basis for the treatment of Class II division 1 malocclusion patients with mandibular retraction.

## Methods

### Sample selection

This retrospective study included samples collected from our hospital. This study was approved by the ethics committee of the Stomatology School of the Air Force Medical University (approval no. KQ-YJ-2023–055). A total of 52 patients diagnosed with Class II division 1 malocclusion from January 2018 to July 2023 were recruited as the study cohort. Three patients were excluded owing to problematic CBCT data. All the patients read and signed an informed consent form to participate in this study [17].

**Fig. 1:**
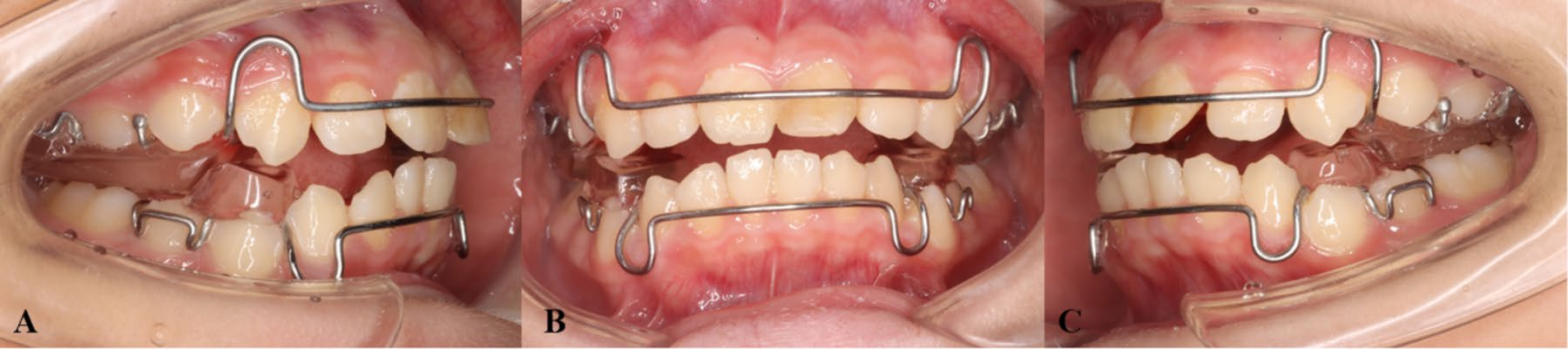
Twin-block (TB) appliance. **A**: right lateral view; **B**: frontal view; **C**: left lateral view

**Fig. 2:**
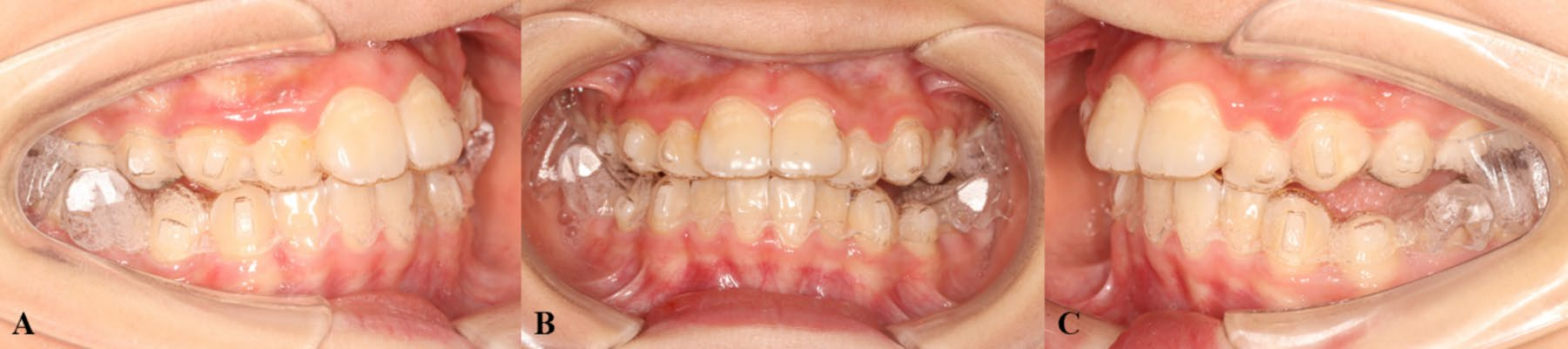
Clear functional aligners. **A**: right lateral view; **B**: frontal view; **C**: left lateral view

The inclusion criteria were as follows:

1. Phase 1 function appliance treatment initiated near pubertal peak, which was defined as cervical vertebral maturation assessment (CVM) stage 2–4 [18, 19]
2. ANB > 5°, SNB ≤ 76°, FMA < 32°, normal or slightly protruding maxilla
3. Class II division 1 malocclusion, overjet > 5 mm, and bilateral class II molar relationships
4. A slight or non-crowded mandibular arch (crowding < 4 mm)
5. Patients were treated with Twin-Block functional appliance or clear functional aligner (Figs. 1 and 2)
6. CBCT scans in good definition and quality (For the accuracy of the experimental data).

The exclusion criteria were as follows:

- 1. History of orthodontic treatment
- 2. History of TMJ disorders
- 3. History of cyst or tumor surgery
- 4. History of cleft lip or cleft palate
- 5. History of systemic disease
- 6. Patients who were lost to follow-up

### Sample size calculation

The sample size was calculated using the G* Power software (version 3.1; Universität Kiel). Considering α = 0.05, β = 0.2, t-test for matched pairs, and an effect size of 0.8 [20, 21], the sample size was calculated as at least 15 patients per group.

### Measurement methods and items

All the participants underwent wide-range CBCT head scans (NewTom AG, Marburg, Germany). CBCT was performed at two time points: T1, at the start of treatment, and T2, at the end of functional therapy. The voxel size of the CBCT is 150 μm and the grey scale is 16-bit. The participants sat on a chair, facing forward, with their head position adjusted such that the Frankfort horizontal plane was parallel to the ground, the median sagittal plane was consistent with the long axis of the fuselage, and the coronal plane was perpendicular to the ground. During scanning, the bilateral molars were tightly occluded in the intercuspation position, with a scanning range, 15 cm × 15 cm, including the upper margin of the orbit to the lower margin of the mandibular body; tube voltage, 120 kV; tube current, 5 mA; scanning duration, 14.7 s, exposure time, 3.6 s, and clarity, 0.3 mm. All the scanning data were saved in the Digital Imaging and Communications in Medicine (DICOM) format on a computer or disc.

**Fig. 3:**
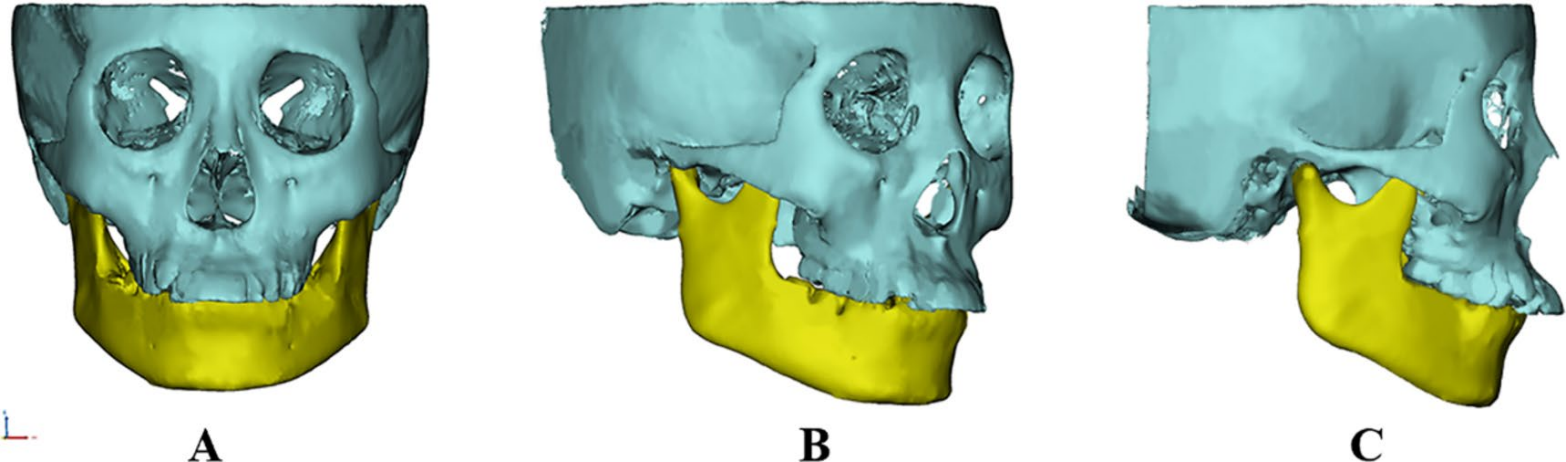
A three-dimensional model of the craniomaxillofacial bones. **A**: Frontal; **B**: 45° Lateral; **C**: Lateral

**Fig. 4:**
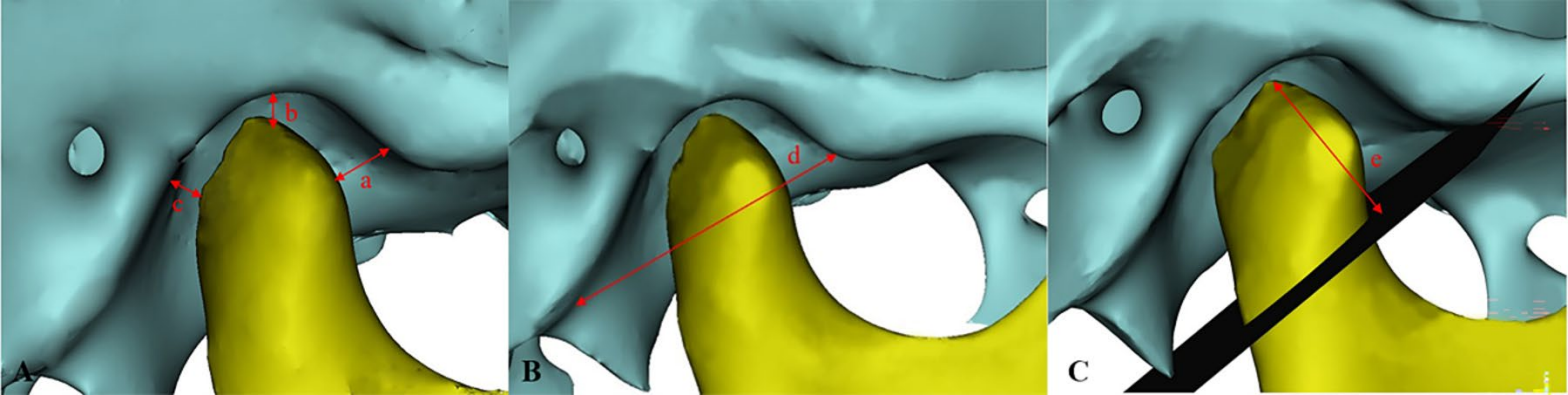
Measurement indices. A: a- Anterior joint space; b- Superior joint space; c- Posterior joint space; B: d- Width of the articular fossa; C: e- Height of the condyle

**Fig. 5:**
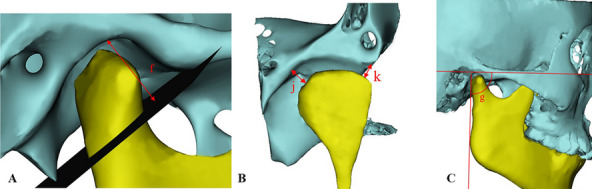
Measurement indices. A: f- Depth of the articular fossa; B: j- Medial joint space; k- Lateral joint space; C: g- Sagittal condylar angle

**Fig. 6:**
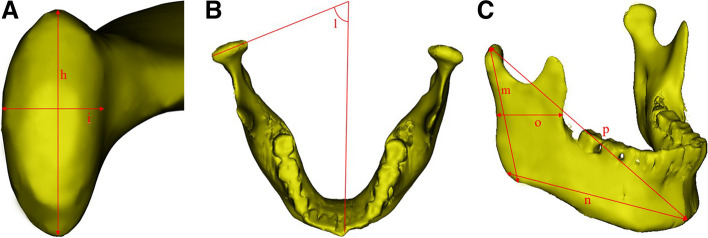
Measurement indices. A:h- Internal and external diameters of the condyle; i- Anterior and posterior diameter of the condyle; B: l- The horizontal condylar angle; C:m- Length of mandibular rami; n- Length of the mandibular body; o-Width of the mandibular rami; p- Mandibular length

**Fig. 7:**
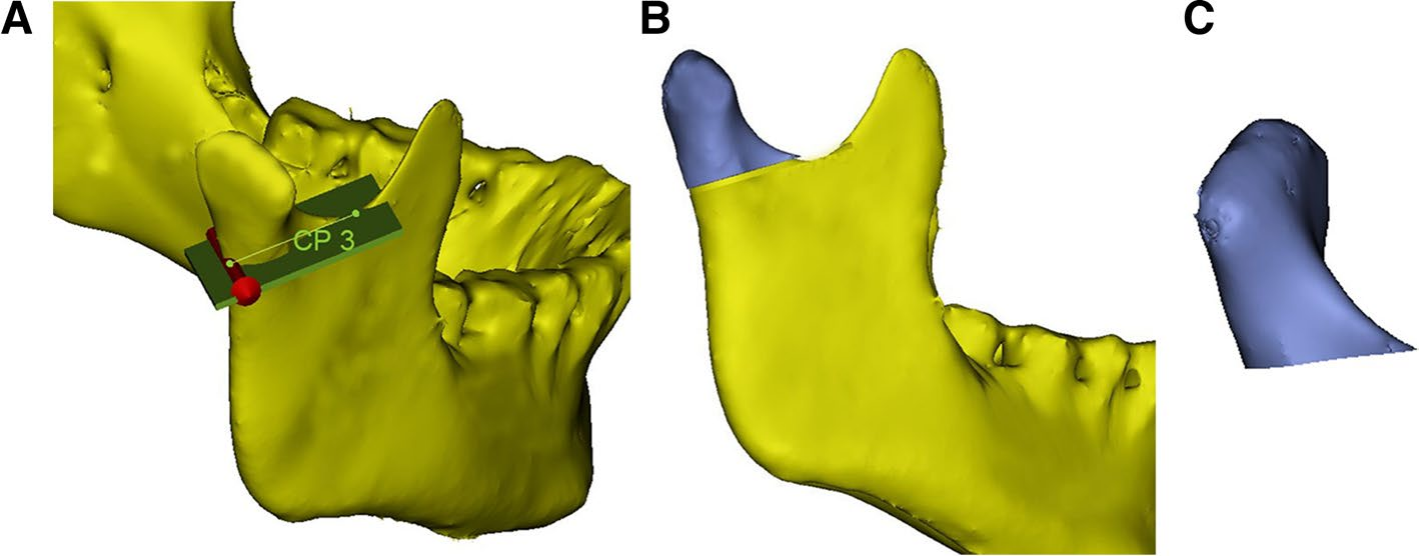
Measurement indices. **A** In the sagittal direction, a vertical line perpendicular to the mandibular ramus was made through the lowest point of the sigmoid notch of the mandible to segment the condyle; **B** a 3D model of the condyle was segmented; **C** a 3D model of the condyle

The CBCT data of the study participants were imported into the MIMICS software (version 21.0; Materialise, Leuven, Belgium) for 3D reconstruction. The head position was adjusted to ensure that the Frankfort plane was parallel to the horizontal plane; the Frankfort, median sagittal, and coronal planes were used as reference planes. We set the threshold and grayscale values, edited the mask and mesh division, used the split mask to separate the upper and lower jaw bones, and used calculate 3D to reconstruct the 3D model of the craniofacial bones (Fig. 3). Smoothing and wrapping were used to trim the rough edges caused by slight movement or imaging artifacts of the study participants during CBCT scanning on the 3D model. The anatomical landmarks of the reconstructed 3D model were then located in the horizontal, sagittal, and coronal directions, and the spatial measurement of each index was performed. The measurement method has been previously reported in literature [22–24], and the measurement items are listed in Table 1 and Figs. 4, 5, 6 and 7.

**Table 1:** Definitions of the measurement indices

Measurement index	Abbreviation	Definition
**Sagittal direction**		
Anterior joint space	AJS	The shortest distance between the most anterior point of the condyle and the posterior point of the joint nodule
Superior joint space	SJS	The shortest distance between the highest point of the condyle and the highest point of the fossa
Posterior joint space	PJS	The shortest distance between the last point of the condyle and the posterior wall of the fossa
Width of the glenoid fossa	WGF	The linear distance between the lowest point of the articular tubercle and the lowest point of the posterior articular process
Depth of the glenoid fossa	DGF	The vertical line from the highest point of the glenoid fossa to the lowest point of the articular tubercle and the lowest point of the posterior articular process
Sagittal condylar angle	SCA	The angle of intersection between the Frankfort plane and the tangential line to the posterior outline of the mandibular ramus
Height of the condyle	HC	The vertical line from the apex of the condyle to the lowest point of the articular tubercle and the lowest point of the posterior articular process
Length of mandibular rami	LMR	The linear distance between the condylar top and ante gonial notch
Length of the mandibular body	LMB	The linear distance between the gonion and gnathion
Width of the mandibular rami	WMR	The linear distance between the anterior and posterior branches of the mandibular branch
Mandibular length	ML	The linear distance between the condylar top and the gnathion
**Coronal direction**		
Medial joint space	MJS	The linear distance from the innermost point of the condyle to the glenoid fossa innermost point of the fovea
Lateral joint space	LJS	The linear distance from the outermost point of the condyle to the glenoid fossa outermost point of the fovea
**Horizontal direction**		
The horizontal condylar angle	HCA	The angle between the condylar long axis (the line between the most medial and lateral points) and the line "tip of the nose, septum of the nose, foramen magnum"
Internal and external diameters of the condyle	IEDC	The linear distance between the most lateral and medial points of the condyle
Anterior and posterior diameters of the condyle	APDC	The linear distance between the anterior and posterior points of the condyle
**Volume of the condyle**	VC	In the sagittal direction, a vertical line perpendicular to the mandibular ramus was made through the lowest point of the sigmoid notch of the mandible to segment the condyle, and the volume of the condyle was measured by MIMICS
**Surface area of the condyle**	SC	The surface area of the condyle was measured by MIMICS using the method described above

Condyle position was assessed according to Pullinger’s method [25] as follows:

$$\mathrm{LR}=\left(\mathrm{posterior\;joint\;space}-\mathrm{anterior\;joint\;space}\right)/\left(\mathrm{posterior\;joint\;space}+\mathrm{anterior\;joint\;space}\right)\times100\%$$

If LR is >12, the condyle is in the articular portion of the fossa; -12 < LR < 12, the condyle is in the middle portion of the fossa; and LR < -12, the condyle is in the posterior portion of the fossa.

### Statistical analysis

Statistical analyses were performed using SPSS (version 25.0; IBM Corp., Armonk, NY, USA). 3D model reconstruction of the craniofacial bones, determination of the anatomical landmarks, and measurement procedures were performed by two researchers over a continuous period of time. Each measurement was performed twice using the same device. Ultimately, the average of four measurements was considered as the measurement result. Both researchers were orthodontists who had performed calibration previously. To evaluate the reproducibility and reliability, the researchers calculated the errors using the Dahlberg [26] formula:

$$D=\sqrt{\sum_{i=1}^{n}\frac{{d}_{i}^{2}}{2n}}$$

where d represents the difference between the two measurements, and n is the sample size for repeated measurements. The results revealed that the measurement error of the data was small, indicating a high level of repeatability. The intraclass correlation coefficient (ICC) of each group was calculated to test the repeatability of the measurements. The results demonstrated decent measurement consistency (ICC = 0.997, 95% CI: 0.991–0.998).

First, the Kolmogorov–Smirnov and Levene tests were used to test the normality and homogeneity of data variance. The test results showed that the data complied with normal distribution and homogeneous variance. The chi-square test was used to compare the gender distribution of patients before treatment with Twin-Block and clear functional aligner, independent sample t-test was used to compare the age, treatment time of the two groups of patients, and TMJ parameters of the two groups of patients before orthodontic treatment to determine the difference before treatment. A paired sample t-test was used to compare the differences in the TMJ parameters between the left and right sides before and after treatment, and the differences in the TMJ parameters before and after treatment. An independent sample t-test was used to compare the difference in the TMJ measurement values between the clear functional aligner and Twin-Block groups before and after treatment. Differences were considered statistically significant at two-sided α = 0.05 and *P* < 0.05.

## Results

### Patient demographics

A total of 49 patients were included in this study, with 25 and 24 patients in the clear functional alignment and Twin block groups, respectively. No significant differences were observed in the sex, age, or treatment duration between the two groups of patients (Table 2). No significant differences were observed in the left and right joint measurement values between the two groups before and after treatment (Tables 3 and 4). The data from the left and right sides of each item were combined. No significant differences were observed in the measurement values between the two groups before treatment (Table 5).

**Table 2:** Comparison of the patient demographics between the two appliance groups

Variable	N	Age (years old)	Treatment duration (months)	Sex	
				**Male**	**Female**
**Clear functional aligners**	25	10.96 ± 0.84	11.82 ± 0.88	12 (48.0%)	13 (52.0%)
**Twin-Block appliance**	24	10.71 ± 0.86	12.15 ± 0.99	12 (50.0%)	12 (50.0%)
***P-value***		0.305	0.229	0.889

**Table 3:** Comparison of the TMJ measurements values on the left and right sides before and after Twin-Block appliance treatment (x ± s, mm)

Variable	Before treatment			After treatment		
	**Left**	**Right**	***P-value***	**Left**	**Right**	***P-value***
AJS	2.73 ± 0.26	2.70 ± 0.23	0.150	2.70 ± 0.19	2.71 ± 0.22	0.708
SJS	2.89 ± 0.23	2.87 ± 0.25	0.460	2.89 ± 0.20	2.87 ± 0.20	0.212
PJS	2.59 ± 0.24	2.55 ± 0.24	0.107	2.62 ± 0.18	2.64 ± 0.19	0.501
MJS	2.87 ± 0.19	2.88 ± 0.21	0.472	2.89 ± 0.28	2.88 ± 0.28	0.528
LJS	2.68 ± 0.20	2.68 ± 0.19	0.967	2.68 ± 0.24	2.65 ± 0.21	0.206
WGF	28.92 ± 0.94	28.95 ± 0.95	0.681	29.45 ± 1.40	29.36 ± 1.45	0.613
DGF	13.42 ± 0.72	13.31 ± 0.76	0.201	14.81 ± 1.11	14.74 ± 1.16	0.571
HC	9.02 ± 0.54	8.99 ± 0.58	0.617	10.98 ± 1.03	10.97 ± 1.03	0.844
SCA (°)	73.30 ± 2.07	73.23 ± 2.05	0.375	73.02 ± 2.61	73.15 ± 2.53	0.347
HCA (°)	73.30 ± 2.61	73.23 ± 2.60	0.129	74.00 ± 2.13	73.77 ± 1.99	0.242
IEDC	16.43 ± 1.36	16.42 ± 1.39	0.821	17.63 ± 1.84	17.49 ± 1.82	0.316
APDC	8.44 ± 0.38	8.39 ± 0.43	0.181	10.25 ± 1.06	10.16 ± 1.16	0.430
VC (mm^3^)	1193.21 ± 144.91	1179.80 ± 158.34	0.146	1277.15 ± 97.26	1263.82 ± 86.64	0.231
SC (mm^2^)	1504.75 ± 109.04	1495.72 ± 107.86	0.069	1607.26 ± 91.96	1629.67 ± 116.22	0.372
LMR	59.71 ± 3.24	59.89 ± 3.29	0.059	64.54 ± 3.43	64.54 ± 3.34	0.988
LMB	78.97 ± 1.89	78.91 ± 2.02	0.510	82.02 ± 1.31	82.13 ± 1.15	0.604
WMR	31.49 ± 2.12	31.61 ± 2.25	0.065	32.53 ± 1.86	32.58 ± 1.85	0.770
ML	112.18 ± 2.69	112.41 ± 2.55	0.095	117.53 ± 3.92	117.46 ± 4.36	0.863

*TMJ* temporomandibular joint, *AJS* anterior joint space, *SJS* superior joint space, *PJS* posterior joint space, *MJS* medial joint space, *LJS* lateral joint space, *WGF* width of the glenoid fossa, *DGF* depth of the glenoid fossa, *HC* height of the condyle, *SCA* sagittal condylar angle, *HCA* horizontal condylar angle, *IEDC* internal and external diameters of the condyle, *APDC* anterior and posterior diameters of the condyle, *VC* volume of the condyle, *SC* surface area of the condyle, *LMR* length of mandibular rami, *LMB* length of the mandibular body, *WMR* width of the mandibular rami, *ML* mandibular length

**Table 4:** Comparison of the TMJ measurement values on the left and right sides before and after clear functional aligners treatment (x ± s, mm)

Variable	Before treatment			After treatment		
	**Left**	**Right**	***P-value***	**Left**	**Right**	***P-value***
AJS	2.76 ± 0.21	2.74 ± 0.20	0.082	2.72 ± 0.19	2.73 ± 0.19	0.570
SJS	2.93 ± 0.17	2.93 ± 0.17	0.638	3.00 ± 0.22	3.01 ± 0.22	0.533
PJS	2.62 ± 0.24	2.60 ± 0.24	0.244	2.69 ± 0.18	2.66 ± 0.20	0.109
MJS	2.87 ± 0.19	2.89 ± 0.20	0.154	2.89 ± 0.19	2.90 ± 0.19	0.500
LJS	2.60 ± 0.25	2.61 ± 0.23	0.591	2.63 ± 0.25	2.65 ± 0.25	0.220
WGF	29.10 ± 1.24	29.15 ± 1.38	0.656	29.48 ± 1.41	29.43 ± 1.28	0.504
DGF	13.63 ± 0.76	13.65 ± 0.81	0.877	15.41 ± 1.02	15.32 ± 1.02	0.113
HC	9.22 ± 0.60	9.22 ± 0.52	0.978	11.75 ± 0.87	11.45 ± 0.83	0.060
SCA (°)	73.74 ± 2.44	73.89 ± 2.49	0.462	73.20 ± 1.99	72.06 ± 2.05	0.096
HCA (°)	72.54 ± 3.01	72.81 ± 3.25	0.443	72.58 ± 3.27	72.57 ± 3.43	0.889
IEDC	16.55 ± 1.93	16.89 ± 2.04	0.074	17.60 ± 1.54	17.64 ± 1.62	0.625
APDC	8.50 ± 0.65	8.52 ± 0.68	0.790	9.36 ± 0.74	9.38 ± 0.80	0.778
VC (mm^3^)	1206.26 ± 260.81	1175.46 ± 228.62	0.393	1258.60 ± 153.97	1244.23 ± 157.33	0.395
SC (mm^2^)	1478.91 ± 201.93	1511.99 ± 196.03	0.094	1606.33 ± 163.27	1610.45 ± 161.10	0.751
LMR	60.22 ± 4.71	60.68 ± 5.05	0.052	63.28 ± 4.25	63.53 ± 4.45	0.084
LMB	79.44 ± 4.20	79.01 ± 4.23	0.211	81.51 ± 4.09	81.42 ± 4.11	0.770
WMR	31.16 ± 2.42	31.14 ± 2.00	0.919	31.68 ± 2.08	31.70 ± 2.06	0.855
ML	113.46 ± 4.85	113.75 ± 4.91	0.128	117.62 ± 3.76	117.74 ± 3.86	0.132

*TMJ* temporomandibular joint, *AJS* anterior joint space, *SJS* superior joint space, *PJS* posterior joint space, *MJS* medial joint space, *LJS* lateral joint space, *WGF* width of the glenoid fossa, *DGF* depth of the glenoid fossa, *HC* height of the condyle, *SCA* sagittal condylar angle, *HCA* horizontal condylar angle, *IEDC* internal and external diameters of the condyle, *APDC* anterior and posterior diameters of the condyle, *VC* volume of the condyle, *SC* surface area of the condyle, *LMR* length of mandibular rami, *LMB* length of the mandibular body, *WMR* width of the mandibular rami, *ML* mandibular length

**Table 5:** Comparison of the TMJ measurements between the two groups before orthodontic treatment (x ± s, mm)

Variable	Clear functional aligners	Twin-Block appliance	T	*P-value*
AJS	2.72 ± 0.25	2.75 ± 0.20	-0.773	0.441
SJS	2.88 ± 0.24	2.93 ± 0.17	-1.168	0.246
PJS	2.57 ± 0.24	2.61 ± 0.24	-0.913	0.363
MJS	2.87 ± 0.19	2.88 ± 0.19	-0.074	0.941
LJS	2.68 ± 0.19	2.61 ± 0.24	1.524	0.131
WGF	28.94 ± 0.94	29.12 ± 1.30	-0.806	0.422
DGF	13.36 ± 0.73	13.64 ± 0.78	-1.800	0.075
HC	9.00 ± 0.56	9.22 ± 0.56	-1.937	0.056
SCA (°)	73.27 ± 2.03	73.82 ± 2.45	-1.208	0.230
HCA (°)	73.26 ± 2.57	72.68 ± 3.11	1.012	0.314
IEDC	16.43 ± 1.36	16.72 ± 1.97	-0.857	0.393
APDC	8.41 ± 0.40	8.51 ± 0.66	-0.848	0.399
VC (mm^3^)	1186.50 ± 150.30	1190.86 ± 243.23	-0.106	0.916
SC (mm^2^)	1500.24 ± 107.39	1495.45 ± 197.66	0.148	0.883
LMR	59.80 ± 3.23	60.45 ± 4.84	-0.780	0.437
LMB	78.94 ± 1.94	79.23 ± 4.18	-0.437	0.663
WMR	31.55 ± 2.16	31.15 ± 2.20	0.896	0.373
ML	112.29 ± 2.60	113.61 ± 4.83	-1.663	0.100

*TMJ* temporomandibular joint, *AJS* anterior joint space, *SJS* superior joint space, *PJS* posterior joint space, *MJS* medial joint space, *LJS* lateral joint space, *WGF* width of the glenoid fossa, *DGF* depth of the glenoid fossa, *HC* height of the condyle, *SCA* sagittal condylar angle, *HCA* horizontal condylar angle, *IEDC* internal and external diameters of the condyle, *APDC* anterior and posterior diameters of the condyle, *VC* volume of the condyle, *SC* surface area of the condyle, *LMR* length of mandibular rami, *LMB* length of the mandibular body, *WMR* width of the mandibular rami, *ML* mandibular length

### Changes in the TMJ before and after treatment with the Twin-Block appliance

After treatment with the Twin-Block appliance, the height, internal and external diameter, anterior and posterior diameter, volume, surface area of the condyle, and depth of the articular fossa increased, while the length of the mandibular rami, length of the mandibular body, width of the mandibular rami, and mandibular length also increased. The average increase in condylar height was approximately 1.97 mm, and the average increase in condylar volume was approximately 83.98 mm^3^. All the above results were statistically significant (Table 6).

**Table 6:** Comparison of the TMJ measurement values before and after treatment in the Twin-Block functional appliance group (x ± s, mm)

Variable	Before treatment	After treatment	*P-value*
AJS	2.72 ± 0.25	2.70 ± 0.21	0.652
SJS	2.88 ± 0.24	2.88 ± 0.20	0.954
PJS	2.57 ± 0.24	2.63 ± 0.18	0.069
MJS	2.87 ± 0.19	2.88 ± 0.28	0.695
LJS	2.68 ± 0.19	2.66 ± 0.23	0.664
WGF	28.94 ± 0.94	29.41 ± 1.41	0.075
DGF	13.36 ± 0.73	14.77 ± 1.12	0.001*
HC	9.00 ± 0.56	10.97 ± 1.02	0.001*
SCA (°)	73.27 ± 2.03	73.08 ± 2.54	0.640
HCA (°)	73.26 ± 2.57	73.89 ± 2.04	0.068
IEDC	16.43 ± 1.36	17.82 ± 1.32	0.001*
APDC	8.41 ± 0.40	10.20 ± 1.10	0.001*
VC (mm^3^)	1186.50 ± 150.30	1270.48 ± 91.37	0.001*
SC (mm^2^)	1500.24 ± 107.39	1618.27 ± 104.29	0.001*
LMR	59.80 ± 3.23	64.54 ± 3.35	0.001*
LMB	78.94 ± 1.94	82.07 ± 1.22	0.001*
WMR	31.55 ± 2.16	32.56 ± 1.84	0.001*
ML	112.29 ± 2.60	117.49 ± 4.10	0.001*

*TMJ* temporomandibular joint, *AJS* anterior joint space, *SJS* superior joint space, *PJS* posterior joint space, *MJS* medial joint space, *LJS* lateral joint space, *WGF* width of the glenoid fossa, *DGF* depth of the glenoid fossa, *HC* height of the condyle, *SCA* sagittal condylar angle, *HCA* horizontal condylar angle, *IEDC* internal and external diameters of the condyle, *APDC* anterior and posterior diameters of the condyle, *VC* volume of the condyle, *SC* surface area of the condyle, *LMR* length of mandibular rami, *LMB* length of the mandibular body, *WMR* width of the mandibular rami, *ML* mandibular length

^*^P < 0.05

### Changes in the TMJ before and after treatment with clear functional aligners

After treatment with clear functional aligners, the height, internal and external diameters, anterior and posterior diameters, volume, surface area of the condyle, superior joint space, and depth of the articular fossa increased, whereas the length of the mandibular rami and mandibular body, width of the mandibular rami, and mandibular length increased. The average increase in condylar height was approximately 2.38 mm, and the average increase in condylar volume was approximately 60.55 mm^3^. All the above results were statistically significant (Table 7).

**Table 7:** Comparison of the TMJ measurement values before and after treatment in the clear functional aligner group (x ± s, mm)

Variable	Before treatment	After treatment	*P-value*
AJS	2.75 ± 0.20	2.72 ± 0.19	0.200
SJS	2.93 ± 0.17	3.01 ± 0.22	0.024*
PJS	2.61 ± 0.24	2.67 ± 0.19	0.057
MJS	2.88 ± 0.19	2.89 ± 0.19	0.183
LJS	2.61 ± 0.24	2.64 ± 0.25	0.069
WGF	29.12 ± 1.30	29.45 ± 1.33	0.149
DGF	13.64 ± 0.78	15.37 ± 1.01	0.001*
HC	9.22 ± 0.56	11.60 ± 0.85	0.001*
SCA (°)	73.82 ± 2.45	73.13 ± 2.00	0.058
HCA (°)	72.68 ± 3.11	72.57 ± 3.32	0.814
IEDC	16.72 ± 1.97	17.62 ± 1.57	0.001*
APDC	8.51 ± 0.66	9.37 ± 0.77	0.001*
VC (mm^3^)	1190.86 ± 243.23	1251.41 ± 154.23	0.004*
SC (mm^2^)	1495.45 ± 197.66	1608.39 ± 160.54	0.001*
LMR	60.45 ± 4.84	63.40 ± 4.31	0.001*
LMB	79.23 ± 4.18	81.46 ± 4.05	0.001*
WMR	31.15 ± 2.20	31.69 ± 2.05	0.020*
ML	113.61 ± 4.83	117.68 ± 3.77	0.001*

*TMJ* temporomandibular joint, *AJS* anterior joint space, *SJS* superior joint space, *PJS* posterior joint space, *MJS* medial joint space, *LJS* lateral joint space, *WGF* width of the glenoid fossa, *DGF* depth of the glenoid fossa, *HC* height of the condyle, *SCA* sagittal condylar angle, *HCA* horizontal condylar angle, *IEDC* internal and external diameters of the condyle, *APDC* anterior and posterior diameters of the condyle, *VC* volume of the condyle, *SC* surface area of the condyle, *LMR* length of mandibular rami, *LMB* length of the mandibular body, *WMR* width of the mandibular rami, *ML* mandibular length

^*^P < 0.05

### Comparison of the measurement value differences before and after treatment between the clear functional aligners and Twin-Block appliance groups

The changes in the TMJ measurement values before and after treatment between the two groups of appliances were as follows. Significant differences were observed in the measurement values of the height of the condyle, anterior and posterior diameters, and length of the mandibular rami.

**Fig. 8:**
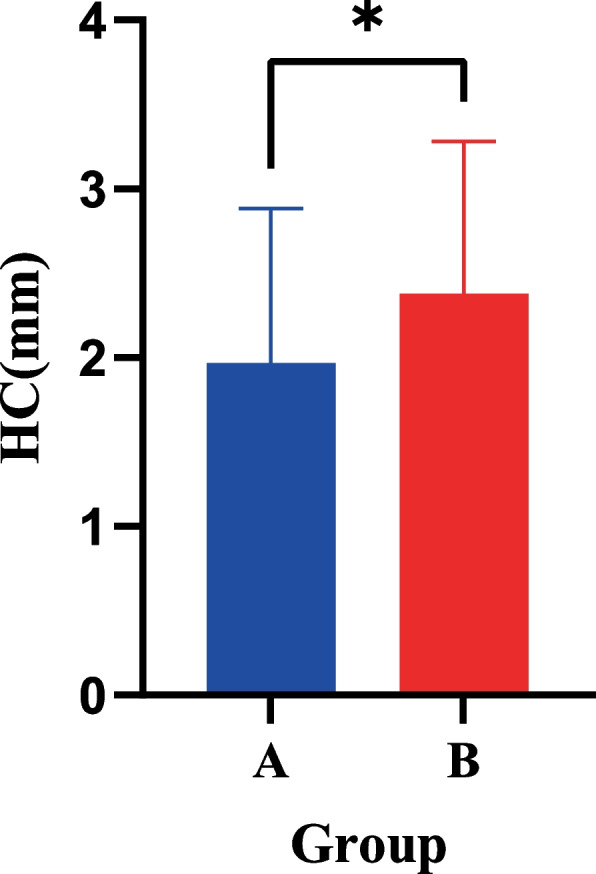
The absolute increase in the condylar height was significantly higher in the clear functional aligners group (**B**) than in the Twin-Block group (**A**) after treatment. *P < 0.05; **P < 0.01; ***P < 0.0001

**Fig. 9:**
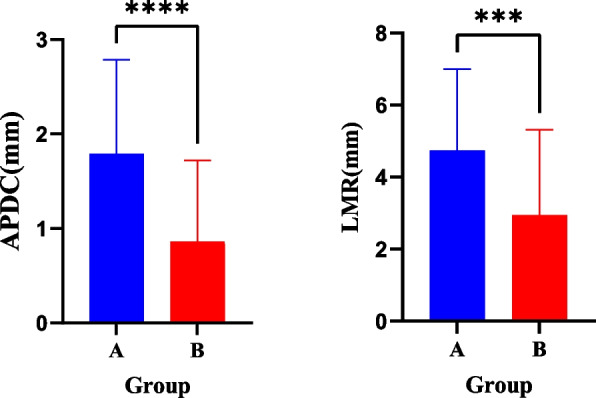
The absolute increase in the anterior and posterior diameters of the condyle and length of mandibular rami were significantly higher in the Twin-Block group (**A**) than in the clear functional aligners group (**B**) after treatment. *P < 0.05; **P < 0.01; ***P < 0.0001

In the clear functional aligner group, the increase in the height of the condyle was greater than that in the Twin-Block appliance group. In the Twin-Block appliance group, the increase in the length of the mandibular rami and anterior and posterior diameters of the condyle were greater than those in the clear functional aligner group (Table 8, Figs. 8 and 9).

**Table 8:** Comparison of the changes in the TMJ measurement values after clear functional aligners and Twin-Block appliance treatments (x ± s, mm)

Variable	Clear functional aligners	Twin-Block appliance	T	*P-value*
AJS	-0.02 ± 0.24	-0.03 ± 0.16	0.341	0.734
SJS	-0.00 ± 0.20	0.08 ± 0.25	-1.829	0.071
PJS	0.06 ± 0.23	0.06 ± 0.22	0.033	0.974
MJS	0.01 ± 0.19	0.02 ± 0.09	-0.194	0.846
LJS	-0.01 ± 0.20	0.03 ± 0.12	-1.325	0.188
WGF	0.47 ± 1.78	0.33 ± 1.59	0.399	0.691
DGF	1.41 ± 1.39	1.73 ± 1.07	-1.282	0.203
HC	1.97 ± 0.92	2.38 ± 0.90	-2.226	0.028*
SCA (°)	-0.18 ± 2.66	-0.68 ± 2.49	0.966	0.337
HCA (°)	0.62 ± 2.32	-0.10 ± 3.07	1.319	0.190
IEDC	1.39 ± 1.03	0.90 ± 1.47	1.902	0.060
APDC	1.79 ± 1.00	0.86 ± 0.86	4.935	0.001*
VC (mm^3^)	83.98 ± 137.46	60.55 ± 139.78	0.836	0.405
SC (mm^2^)	118.23 ± 110.73	112.94 ± 124.34	0.222	0.825
LMR	4.74 ± 2.26	2.95 ± 2.37	3.829	0.001*
LMB	3.14 ± 1.57	2.24 ± 2.94	1.878	0.063
WMR	1.01 ± 1.32	0.53 ± 1.57	1.618	0.109
ML	5.20 ± 3.55	4.07 ± 3.62	1.548	0.125

*AJS* anterior joint space, *SJS* superior joint space, *PJS* posterior joint space, *MJS* medial joint space, *LJS* lateral joint space, *WGF* width of the glenoid fossa, *DGF* depth of the glenoid fossa, *HC* height of the condyle, *SCA* sagittal condylar angle, *HCA* horizontal condylar angle, *IEDC* internal and external diameters of the condyle, *APDC* anterior and posterior diameters of the condyle, *VC* volume of the condyle, *SC* surface area of the condyle, *LMR* length of mandibular rami, *LMB* length of the mandibular body, *WMR* width of the mandibular rami, *ML* mandibular length

^*^P < 0.05

The condylar positions in the two groups before and after orthodontic treatment were calculated according to Pullinger’s method [25]. Before treatment, approximately 12.0% of the condyles in the clear functional aligner group and 12.5% of the condyles in the Twin-Block appliance group were in the posterior portion of the articular fossa. After treatment, the condyles in both the groups were in the neutral position of the articular fossa (Table 9).

**Table 9:** Comparison of the condylar position in the articular fossa before and after orthodontic treatment (n, %)

Variable	Clear functional aligners				Twin-Block appliance			
	**Before treatment**	**After treatment**	**χ2**	**P**	**Before treatment**	**After treatment**	**χ2**	***P-value***
**N**	50	50	6.383	0.012*	48	48	6.400	0.011*
**Condyle in anteposition**	0	0			0	0		
**Condyle in middle**	44 (88.0%)	50 (100%)			42 (87.5%)	48 (100%)		
**Condyle in retroposition**	6 (12.0%)	0			6 (12.5%)	0		

^*^P < 0.05

## Discussion

Mandibular retrusion or mandibular hypoplasia, which is commonly observed in most patients with Class II division 1 malocclusion, seriously affects oral function and facial esthetics. Twin-Block and clear functional aligners are commonly used in clinical practice for the management of mandibular retrusion in adolescent patients. Many studies have demonstrated mandibular advancement treatment to be closely related to TMJ [27–32]; This study used a three-dimensional spatial measurement method to directly measure the TMJ on the reconstructed 3D model, aiming to compare the effects and differences on TMJ after treatment with Twin-Block and clear functional aligners.

### Research methods for TMJ

The TMJ is among the most complex joints in the human body. Due to its deep location, TMJ conditions are often diagnosed and analyzed using imaging techniques. CBCT is increasingly used in the basic diagnosis and treatment of oral and maxillofacial regions owing to its advantages, such as low radiation dose, high imaging accuracy, few artifacts, accurate reflection of the condyle shape and position, and 3D reconstruction [33]. Bayram et al. reported the reliability and accuracy of volumetric analysis for evaluating condylar changes using CBCT. The measured condylar volume was consistent with the actual volume when the cross-sectional thickness was 0.3–0.9 mm [34]. Many researchers have used CBCT to evaluate the TMJ in various patients with malocclusions and compare the changes in the TMJ before and after orthognathic surgery [35–37].

In our study, CBCT and MIMICS software were integrated to analyze the TMJ three-dimensionally, thereby avoiding the shortcomings of previous two-dimensional imaging measurements. The three-dimensional spatial measurement method can measure the three-dimensional distance between two points [16]. Simultaneously, the data measured on the 3D model can more accurately reflect the real 3D characteristics of the patient’s TMJ growth.

### Condyle growth and displacement

The condyle is one of the growth centers of the mandible during the growth phase in adolescents. The fibrocartilage covering its surface has strong remodeling and repair capacity, which provides a physiological basis for using functional orthodontic appliances to promote condylar growth and change the position of the mandible [38]. Fan et al. analyzed the morphology of the condyle before and after treatment with functional orthodontic appliances in 20 adolescent patients with Class II malocclusion and found that the sagittal length of the condyle increased by 1.5–3.0 mm after treatment [39]. Wei et al. also found that the use of functional orthodontic appliances in adolescent patients with Class II malocclusion resulted in the adaptive growth in the upper and posterior parts of the condyle [40].

Regardless of the use of the Twin-Block appliance or clear functional aligners, the height, internal and external diameter, anterior and posterior diameter, volume, and surface area of the condyle all increased, and the differences between the two groups before and after treatment were statistically significant. This indicates that both the functional appliances can promote condylar growth. The increase in the condylar measurement indices observed in this study was attributed to a combination of functional orthodontic treatment and natural growth. A meta-analysis revealed that the annual mandibular growth of the experimental group treated with functional appliances was 1.79 mm higher than that of the untreated control group [41]. Therefore, we can further infer that functional orthodontic treatment of mandibular retrognathism in Class II division 1 patients may stimulate condylar reconstruction by promoting mandibular growth rather than merely moving the mandible forward.

Ruf et al. found that functional orthodontic appliances can increase the vertical growth of the condyle by 1.5 times compared with the non-treatment group [31]. Pancherz et al. found that after 7.5 months of treatment with functional orthodontic appliances, although the condyle grew in both the sagittal and vertical directions, growth was mainly concentrated in the vertical direction [42]. In our study, the use of Twin-Block appliances increased the average height of the condyle by approximately 1.97 mm, while the anteroposterior diameter increased by approximately 1.79 mm; The use of clear functional aligners increased the average height of the condyle by approximately 2.38 mm, while the anteroposterior diameter increased by approximately 0.86 mm. This indicates that, in terms of growth direction, the increase in the condylar height with both appliances was greater than the increase in the anterior–posterior diameter, and the difference was statistically significant, which is consistent with the findings by Ruf and Pancherz. Compared with treatment with Twin-Block, the condylar height of clear functional aligners increased more significantly, while the Twin-Block appliance was more effective in increasing the anteroposterior diameter of the condyle.

### Changes of the articular fossa

Under normal circumstances, the articular fossa grows posteriorly and downward. After functional anterior mandibular displacement, the articular fossa grew in the opposite direction; thus, it moved forward to accommodate the anterior movement of the condyle. LeCornu et al. found that functional orthodontic appliances stimulated the growth and reconstruction of the articular fossa, and that the position of the articular fossa was more anterior than before treatment, resulting in a more forward mandibular position [43]. Ruf et al. found that after anterior mandibular displacement treatment, the articular fossa underwent reconstruction, most of which was concentrated in the posterior part and at the top of the articular fossa [44]. In our study, no statistically significant change was observed in the width of the articular fossa after treatment with the two functional appliances. Although the height of the articular fossa increased, reconstruction of the articular fossa could not be confirmed. The reasons for the analysis are as follows: First, the articular fossa undergoes intraperiosteal osteogenesis, which is not obvious on imaging. Second, the articular fossa grows backward and downward, whereas the functional appliance shifts forward; these two may have a counteracting effect. Finally, articular fossa osteogenesis is an intramembranous osteogenesis in the mesenchymal cell aggregation area, which is different from the endochondral osteogenesis of the condyle, and the remodeling of the articular fossa lags behind that of the condyle [45].

### Changes in the condylar position

Controversy still exists over the changes in the condylar position after functional orthodontic mandibular advancement. Spagnuolo et al. found that after functional orthodontic treatment, both the condyle and articular fossa underwent adaptive reconstruction, and no significant change was observed in the relative position of the articular fossa and condyle [46]. Kanon et al. found that the condyle position in 75% of the patients after treatment was more forward than that in the control group, and the condyle had different degrees of forward movement relative to the articular fossa [47]. In our study, after functional orthodontic treatment, the retrodisplaced condyle moved to the middle position of the articular fossa, whereas the rest of the condylar positions did not change significantly.

### Changes in the TMJ space

Our study found no statistically significant differences in the anterior, upper, and posterior joint spaces before and after treatment in the Twin-Block group. There were no significant changes in the anterior and posterior joint spaces after treatment in the clear functional alignment group. However, a comparison of the change in the joint space before and after treatment in the two groups showed that the anterior joint space decreased and the posterior joint space increased, indicating that the condyle may have a trend of anterior-to-lower displacement in the articular fossa. The superior joint space in the clear functional aligner group increased significantly after treatment, indicating that the condyle may have a slight tendency to move forward and downward upon treatment with clear functional aligner. We speculate that this may be because it controls the vertical height of the molars and anterior teeth, and adjusts the occlusal plane [48].

### Changes in the mandibular ramus

Villegas et al. found that the length of the mandibular ramus significantly increased after Twin-Block treatment [49]. Some researchers believe that under orthodontic force, the mandibular ramus in Class II Division 1 malocclusion cases changes most significantly [50]. A statistically significant difference was observed in the length of the mandibular rami between the two groups after treatment with the functional appliances in our study. The increase in the length of the mandibular ramus in the Twin-Block group after treatment was higher than that in the clear functional aligner group, indicating that the Twin-Block has unique advantages in promoting the growth of the mandibular ramus, which could be related to the materials of the two orthodontic appliances [7]. The resin pad of the Twin-Block device located on the occlusal plane has higher strength; therefore, it can provide sufficient traction stimulation to the masseter muscle group for inducing the growth direction of the mandibular ramus and finally promote growth of the condyle. However, the membrane of a clear functional aligner is soft and prone to deformation. Therefore, an enhanced membrane or resin reinforcement block can be used to enhance the strength of the locking structure when using a clear functional aligner [10].

### Changes in the body of the mandible

Whether the mandibular body changes following functional correction is still controversial. Baysal et al. believe Twin-Block to have no significant effect on the mandibular length [51]. However, other researchers believe that Twin-Block promotes the growth of both the length of the mandible and the mandibular ramus [52]. Ghodke et al. observed that the length of the mandibular body significantly increased following treatment with functional orthodontic appliances [53]. In this study, both groups demonstrated a statistically significant increase in the length of the mandibular body after treatment, which is consistent with the findings of the abovementioned studies.

As our study lacked a control group, the influence of the patients' own growth on the experimental results cannot be ruled out. However, in a clinical experimental orthodontic study, establishing a control group comprising untreated patients is challenging. Because the control and treatment group patients need to be followed up and reviewed simultaneously and undergo CBCT scans, ensuring the acceptance and cooperation of the control group patients and their parents is challenging. Moreover, for patients with Class II Division 1 malocclusion with growth potential, ethics dictate mandibular growth stimulation; thus, denying potential treatment intervention to patients for inclusion in the study control group is ethically challenging.

This study is limited by its small sample size. We aim to increase the sample size to analyze the characteristics of TMJ reconstruction after functional orthodontic appliance treatment in future studies. Simultaneously, long-term follow-up should be conducted in patients who have completed treatment to track the stability of TMJ reconstruction.

## Conclusion

1. The volume surface area and height of the condyles increased after Twin-Block appliance or clear functional aligner treatment, indicating that the condyles may have undergone adaptive remodeling.
2. In terms of the growth direction, the increase in the condyle in the vertical direction was greater than that in the sagittal direction after treatment with the two functional appliances. Clear functional aligners may be superior to the Twin-Block in promoting vertical growth of the condyle, while Twin-Block appliances have more advantages in promoting the sagittal growth of the condyle.
3. After treatment with the two functional appliances, remodeling of the articular fossa was not significant.
4. After the two functional appliance treatments, the retrodisplaced condyle moved to the middle position of the articular fossa, while the rest of the condylar position did not change significantly.
5. Both types of appliances can effectively promote vertical and sagittal growth of the mandible. The Twin-Block has a unique advantage of promoting the growth of the mandibular ramus, which may be related to the material of the appliance.

## Data Availability

The datasets used and analyzed in the current study are available from the corresponding author upon reasonable request.

## Abbreviations

TMJ: Temporomandibular joint
3D: Three-dimensional
CBCT: Cone-beam computed tomography
DICOM: Digital imaging and communications in medicine
ICC: Intra-class correlation coefficient
